# NF-κB RelA regulates temporal oligodendrocyte differentiation in the postnatal brains

**DOI:** 10.3389/fncel.2025.1622874

**Published:** 2025-07-22

**Authors:** Kamonrapat Sompub, Norihisa Bizen, Albert S. Baldwin, Hirohide Takebayashi

**Affiliations:** ^1^Division of Neurobiology and Anatomy, Graduate School of Medical and Dental Sciences, Niigata University, Niigata, Japan; ^2^UNC Lineberger Comprehensive Cancer, University of North Carolina at Chapel Hill, Chapel Hill, NC, United States; ^3^Department of Biology, University of North Carolina at Chapel Hill, NC, United States; ^4^Center for Coordination of Research Facilities (CCRF), Niigata University, Niigata, Japan; ^5^Center for Anatomical Studies, Graduate School of Medicine, Kyoto University, Kyoto, Japan

**Keywords:** oligodendrocyte, differentiation, RelA/p65, NF–κB transcription factor, Splicing

## Abstract

The NF-κB signaling pathway responds to a diverse range of cytokines and extracellular stresses, regulating immune responses, inflammation, cell proliferation, and cell death. However, the requirement of NF-κB in oligodendrocyte development and differentiation remains debatable. In this study, we generated conditional knockout mice of the *RelA* gene in the oligodendrocyte-lineage cells, which encodes a major subunit of NF-κB, and assessed its impact on oligodendrocyte differentiation. In *RelA* cKO mice, we observed a transient delay of oligodendrocyte differentiation in the postnatal cerebral cortex, albeit in a spatially and temporally restricted manner. Similarly, in the primary cultured oligodendrocyte differentiation model, the loss of *RelA* resulted in impaired terminal differentiation. Transcriptome analysis revealed a significant downregulation of numerous oligodendrocyte-related genes, including predicted NF-κB target genes. Furthermore, a comprehensive splicing analysis identified aberrant alternative splicing of *Plp1*, a most abundant and key gene involved in myelin sheath formation. These findings suggest that NF-κB/RelA contributes to the temporal and special control of oligodendrocyte development and differentiation in the postnatal brains. Our results highlight a previously underappreciated role of NF-κB in oligodendrocyte biology and encourage a re-evaluation of its physiological significance in the glial lineage.

## 1 Introduction

Oligodendrocytes are a type of glial cell in the central nervous system (CNS) that generate myelin sheaths around axons, thereby enabling saltatory conduction and contributing to axonal protection and metabolic support (Nave, 2010; Lee et al., 2012; Mot et al., 2018). Oligodendrocytes originate from neural stem cells through the generation of oligodendrocyte precursor cells (OPCs), which subsequently undergo a series of well-coordinated steps involving differentiation and maturation to become myelinating oligodendrocytes eventually (Rowitch and Kriegstein, 2010; Takebayashi and Ikenaka, 2015). This developmental process is governed by a complex interplay of intrinsic and extrinsic factors, including transcription factors, epigenetic regulators, and various signaling molecules (He and Lu, 2013; Zuchero and Barres, 2013; Bercury and Macklin, 2015; Elbaz and Popko, 2019).

Nuclear factor kappa B (NF-κB) is a family of protein complexes that function as transcription factors, regulating the expression of genes involved in various biological processes, including immune responses, inflammation, cell proliferation, development, and carcinogenesis (Baldwin, 2001; Dolcet et al., 2005). The NF-κB family consists of five subunits—p50, p52, RelA (p65), RelB, and c–Rel—which form homo- and heterodimers to exert their transcriptional activity (Karin and Lin, 2002; Yan and Greer, 2008). In its inactive state, NF–κB is sequestered in the cytoplasm by inhibitor proteins known as Inhibitor of Kappa B (IκB), which prevent its activation (Steinbrecher et al., 2008). Upon external stimuli such as cytokines, pathogens, or cellular stress, the IκB kinase (IKK) complex phosphorylates IκB, leading to its ubiquitin-dependent degradation. This degradation releases NF–κB, allowing it to translocate into the nucleus and activate the transcription of target genes. NF–κB signaling is a fundamental intracellular signaling pathway that plays a central role in regulating systemic inflammatory responses. The function of NF–κB in pathological conditions has been extensively studied across various tissues and cell types. However, its physiological roles in development, homeostasis, and normal cellular function remain poorly understood.

In the CNS, the physiological significance of NF–κB signaling in development and maturation has been reported in various neural cell types. In embryonic mouse brains, NF–κB signaling is suggested to contribute to maintaining neural progenitor cells in an undifferentiated state (Yamanishi et al., 2015). In the postnatal brain, NF-κB promotes dendritic spine formation and excitatory synapse development (Boersma et al., 2011). In astrocytes, NF-κB has been implicated in the central regulation of metabolism, including glucose homeostasis, blood pressure, and body weight control (Zhang et al., 2017). Furthermore, in microglia, NF-κB signaling plays a crucial role in maintaining neuronal excitability and synaptic plasticity (Kyrargyri et al., 2015). In contrast, the involvement of NF-κB signaling in oligodendrocyte development and differentiation remains debatable. Clinical studies have reported that patients with increased copy numbers of *IKBKG* gene, which encodes NF-κB essential modulator (NEMO), exhibit reduced NF-κB signaling, accompanied by abnormal myelination, morphological abnormalities in the developing brain, and mild intellectual disability (Philippe et al., 2013). However, analyses using mouse models deficient in key NF–κB components have suggested that NF–κB signaling does not impact on oligodendrocyte differentiation. For instance, mice with CNS–specific deletion of *RelA*, a major NF-κB subunit, do not exhibit significant abnormalities in oligodendrocyte numbers or myelin structure (Kretz et al., 2014). Similarly, the loss of IκB kinase beta (*IKKβ*), a key activator of NF-κB signaling, has no effects on oligodendrocyte development and differentiation (Raasch et al., 2011).

A critical limitation of many previous studies is the predominant use of conventional knockout mice or conditional knockout mice employing the *Nestin-Cre* system (Tronche et al., 1999), which result in the deletion of NF–κB signaling across all CNS cell types from early embryonic stages. This widespread deletion makes it difficult to evaluate the cell-autonomous role of NF–κB signaling, specifically in oligodendrocytes. Therefore, further studies using oligodendrocyte-specific gene deletion models are necessary to elucidate the precise role of NF-κB signaling in oligodendrocyte differentiation.

In this study, we generated a mouse model in which the *RelA* gene was specifically deleted in oligodendrocyte lineage cells and performed a detailed phenotypic analysis. In *RelA*-deficient mice, oligodendrocyte differentiation in the cerebral cortex was delayed at postnatal day 14 (P14); however, this delay was largely resolved by P21. Furthermore, in an *in vitro* oligodendrocyte culture system, *RelA* deficiency resulted in cell-autonomous inhibition of oligodendrocyte differentiation. Transcriptome analysis revealed that in *RelA*-deficient mice, the expression of oligodendrocyte-associated genes predicted to be direct targets of RelA was significantly downregulated. Additionally, splicing alterations of the proteolipid protein 1 (*Plp1)* gene, which encodes a major myelin protein, were detected. These findings suggest that RelA plays a limited but critical role in regulating the timing of oligodendrocyte differentiation and may also contribute to oligodendrocyte maturation through transcriptional regulation and alternative splicing.

## 2 Materials and methods

### 2.1 Animals

We used *Cnp-iCre* knockin mice (MGI:6865676; Bizen et al., 2022) and *RelA^flox/flox^* mice (MGI:3775205; Steinbrecher et al., 2008). We generated conditional knockout (cKO) mice by crossing female *Cnp-iCre*; *RelA*^*flox*+^ mice with male *RelA^flox/flox^* mice. Unless otherwise specified, *RelA^flox/flox^* or *RelA^flox/+^* mice were used as control animals. The day of birth was designated as postnatal day zero (P0). Male and female mice aged P14 and P21 were used. The mice were kept at 22°C ± 2°C and 60% humidity in a 12–h light/12–h dark cycle with ad libitum feeding. The Animal Research Committees of Niigata University approved all methods, and the Guide for the Care and Use of Laboratory Animals of the Institute for Laboratory Animal Research was followed.

### 2.2 Genotyping

Mice were genotyped by polymerase chain reaction (PCR) using primers that amplifies the *RelA^flox^* allele and *Cnp-iCre* allele. The *RelA^flox^* and *RelA^wt^* alleles was detected using the primers RelA-loxF; 5′–CGA CTT TGG GTT GGA GGG TTA CAG AAG GC–3′; RelA-loxR; 5′–TGG TCT GGA TTC GCT GGC TAA TGG C–3′, which amplify 450 bp fragments from *RelA^wt^* allele and 510 bp fragments from *RelA^flox^* allele. The wild-type *Cnp* allele was detected using the primers Cnp-F; 5′–GAA CTC GGC CAG AGA CTA GGG TGT–3′ and Cnp-R; 5′–CCG CGC AGG ATG AAT AGC GTC TTG CAC TCG–3′, which amplify 250 bp fragments. The *Cnp-iCre* allele was detected using the primers Cnp-F; 5′–GAA CTC GGC CAG AGA CTA GGG TGT–3′ and Cnp-iCreR; 5′-CAG GAA GGC CAG GTT CCT GAT GTC–3′, which amplify 658 bp fragments. We used Quick Taq HS DyeMix (Toyobo, Japan) and the PCR machines (PCR Thermal Cycler Dice Gradient, TaKaRa Bio; C1000 Touch Thermal Cycler, Bio-Rad). The PCR detecting *RelA^flox^* and *RelA^wt^* allele was performed (95°C for 2 min, 35 cycles of 95°C for 25 s, 60°C for 25 s, and 72°C for 40 s, followed by 72°C for 3 min). The PCR detecting *Cnp-iCre* allele was performed under the following condition (94°C for 2 min, 32 cycles of 94°C for 30 s, 60°C for 30 s, and 72°C for 1 min, followed by 72°C for 3 min).

### 2.3 Preparation of tissue sections

Cryosections were prepared as previously reported (Horie et al., 2016) and used for histological study. Mice were anesthetized with a lethal dosage of sodium pentobarbital (125 mg/kg body weight) and perfused transcardially with approximately 2 mL of 0.01 M phosphate buffered saline (PBS), followed by 20–25 mL of 4% (w/v) paraformaldehyde in PBS (4% PFA). Brains and spinal cords were removed, soaked in 4% PFA overnight at 4°C, and then incubated with 20% sucrose in PBS overnight at 4°C. On the next day, samples were embedded in OCT compound (Sakura FineTek). Coronal sections (12 μm) were cut using a cryostat (HM525 NX, PHC Corporation).

### 2.4 Immunohistochemistry

Cryosections were washed with PBS for 15 min. Then, sections were treated with microwave irradiation (500 W for 5 min) in 10 mM citrate buffer (pH 6.0) for antigen retrieval and then cooled to room temperature. After rinsing with PBS for 15 min, the sections were incubated with the primary antibodies in phosphate buffered saline with Tween 20 (PBST) (0.1% TritonX–100 in PBS) with 0.5% skimmed milk (Wako, Osaka, Japan) overnight at 4°C. After washing with PBS for 15 min, the sections were incubated in PBST with 0.5% skimmed milk containing secondary antibodies [peroxidase-labeled anti-mouse, anti-rabbit IgG (1:200, MBL, Nagoya, Japan) or peroxidase-labeled anti-rat IgG (1:200, DAKO)], for 1 h at 37°C, and the rinsed in distilled water for 15 min. Immunoreactivity was visualized in 50 mM Tris buffer (pH 7.4) containing 0.01% diaminobenzidine tetrahydrochloride (DAB) and 0.01% hydrogen peroxide for 2–10 min at 37°C. Sections were dehydrate through ethanol and xylene and then coverslipped.

For fluorescent immunohistochemistry (IHC), sections were initially rinsed with PBS three times for 5 min each and treated 10 mM citrate buffer (pH 6.0) for 5 min at 100°C. After washing with PBS three times for 5 min each, the sections were incubated with 10% goat serum in PBST for permeabilization and blocking. Afterward, the sections were further incubated with primary antibodies in PBST containing 10% goat serum overnight at 4°C. After rinsing with PBST for 15 min, the sections were incubated with secondary antibodies for 60 min at room temperature. After rinsing with PBS for 15 min, the sections were incubated with 4′,6–diamidino–2–phenylindole (DAPI, 1 μg/mL, Dojindo) for 10 min at room temperature, then washed with PBS twice for 5 min each. The images were collected using Olympus microscope (BX53, Olympus) and digital camera system (DP74, Olympus). Working dilutions and sources of antibodies used in this study are listed in Table 1.

**TABLE 1:** Antibodies for immunohistochemistry and immunocytochemistry.

Antibody	Dilution	Cat#	Company or reference
Mouse CC1	1:500	OP80	Millipore
Mouse anti–CNPase	1:1500	836404	BioLegend
Rabbit anti–GFAP	1:100	442251	Nichirei
Rabbit anti–Iba1	1:1,000	019-19741	Wako
Rat anti–MBP	1:300	Ab7349	Abcam
Mouse anti–NeuN	1:1,000	834501	BioLegend
Rabbit anti-NF–κB (p65)	1:500	8242	Cell Signaling Technology
Mouse anti–Olig1	1:200	73–180	NeuroMab
Rat anti–PDGFRα	1:300	135910	BioLegend
Rat anti–PLP	1:300	AA3	Yamamura et al., 1991

### 2.5 *In situ* hybridization

The sections were rinsed with PBS for 10 min. The sections were fixed in 4% PFA for 20 min. Following washing with PBS twice for 5 min each, the sections were treated with 1 μg/ml proteinase K in Tris-based buffer [50 mM Tris–HCl [pH 7.6], 5 mM Ethylenediaminetetraacetic acid (EDTA)] for 10 min, and then rinsed in PBS for 5 min. After fixation in 4% PFA in PBS for 15 min and acetylation in 0.1 M triethanolamine (pH 8.0) containing 0.25% acetic anhydride for 10 min, the sections were prehybridized for 2.5 h at 65°C with a hybridization solution [50% formamide, saline sodium citrate (SSC) (0.15 M NaCl, 0.015 M sodium citrate in diethylpyrocarbonate-treated water), 0.2 mg/mL yeast tRNA, 0.1 mg/mL heparin, 1 × Denhardt’s solution, 0.2% Tween 20, 0.1% CHAPS, and 5 mM EDTA]. The sections were then incubated with a hybridization solution containing diluted Digoxigenin (DIG)-labeled RNA probe overnight at 65°C.

The hybridized sections were washed with 1 × SSC and 50% formamide twice for 30 min at 65°C, and then washed with 0.1 × SSC once at 65°C for 30 min. The sections were washed twice in maleic acid buffer [0.1 M maleic acid (pH 7.5), 0.15 M NaCl and 0.1% Tween 20] for 30 min at room temperature and incubated with alkaline phosphatase-conjugated sheep anti-DIG antibody (1:2000, Roche Diagnostics, Manheim, Germany) in overnight at 4°C. They were then washed in maleic acid buffer three times for 30 min each, and incubated with the color development solution [50 μg/mL 4-nitro blue tetrazolium chloride and 175 μg/mL 5–bromo–4–chloro–3–indolyl-phosphate (Roche Diagnostics)] in alkaline phosphatase buffer [0.1 M Tris–HCl (pH 9.5), 0.05 M MgCl_2_, 0.1 M NaCl, and 0.1% Tween 20] for 3–10 h in the dark. The following probes generated from mouse cDNA were used: *Plp* (Kagawa et al., 1994); Myelin basic protein (*Mbp*) (Genbank accession number: BC004704, nt 544–1975); Platelet-derived growth factor receptor alpha (*Pdgfrα*) (EST clone, AI098416, Invitrogen); G protein-coupled receptor 17 (*Gpr17*) (BC070439, nt 270–945); Ectonucleotide pyrophosphatase/phosphodiesterase 6 (*Enpp6*) (NM_177304, nt 375–1211); *Myelin oligodendrocyte glycoprotein* (*Mog*) (NM_010814, nt 324–1112). Sections were counterstained by nuclear fast red (Fluka).

### 2.6 Reverse transcription-quantitative PCR (RT–qPCR) and semi-quantitative RT–PCR

RT-qPCR was performed as described previously (Hayakawa-Yano et al., 2017) with minor modification. Briefly, total RNA was extracted from mouse cerebral cortices at postnatal day 14 (P14) using RNeasy Mini Kit (Qiagen). One microgram total RNA was reverse transcribed by SuperScript III First-Strand Synthesis System (Thermo Fisher Scientific). The RT-qPCR was performed using StepOnePlus real-time PCR detection system (Applied Biosystems). The results were obtained by the ΔΔCt method. The mRNA levels of interested genes were normalized to the mRNA level of the house-keeping gene *Actb*. Semi-quantitative RT-PCR for splicing variants detection was performed under the following conditions using PCR Thermal Cycler Dice (TaKaRa Bio): *Plp1 Ex2*–*4* (95°C for 1 min, 28 cycles of 95°C for 20 s, 60°C for 30 s, and 72°C for 30 s, followed by 72°C for 2 min), pleckstrin homology like domain, family B, member 1 (*Phldb1*) *Ex20*–*22* and SWI/SNF related BAF chromatin remodeling complex subunit B1 (*Smarcb1*) *Ex1*–*3* (95°C for 1 min, 31 cycles of 95°C for 20 s, 60°C for 30 s, and 72°C for 30 s, followed by 72°C for 2 min). Primers used for RT-qPCR and semi-quantitative RT-PCR were described in Table 2.

**TABLE 2:** Primers for RT-qPCR and semi-quantitative RT-PCR.

Primer name	Sequence forward (5′–3′)	Sequence reverse (5′–3′)
*Actb*	GGCTGTATTCCCCTCCATCG	CCAGTTGGTAACAATGCCATGT
*Cpm*	CCCAGTGCTTTGAAATT ACCCT	TGTTATCGTTCCAAAAGA GCGG
*Enpp6*	CAGAGAGATTGTGAAC AGAGGC	CCGATCATCTGGTGGACCT
*Gfap*	CGGAGACGCATCACCTCTG	AGGGAGTGGAGGAGTCATTCG
*Gjc2*	TCCACAATCATTCCACCTTCG	CAGAAGCGCACATGAGACAG
*Gpr17*	CACCCTGTCAAGTCCC TCAAG	GTGGGCTGACTAGCAGTGG
*Aif1 (Iba1)*	ATCAACAAGCAATTCCTC GATGA	CAGCATTCGCTTCAAGGACATA
*Mbp*	AATCGGCTCACAAGGG ATTCA	TCCTCCCAGCTTAAAGATT TTGG
*Mog*	ACCTCTACCGAAATGGC AAGG	TCACGTTCTGAATCCTAAGGGT
*Olig1*	TCTTCCACCGCATCCCTTCT	CCGAGTAGGGTAGGATAA CTTCG
*Olig2*	TCCCCAGAACCCGATGA TCTT	CGTGGACGAGGACACAGTC
*Padi2*	AGATGATCCTGCGCA CCAAA	GCCAAAGAACGGGTTCTCCA
*Pdgfrα*	AGAGTTACACGTTTGAG CTGTC	GTCCCTCCACGGTACTCCT
*Phldb1 Ex20*–*22*	CTGAAGCGCACCCTTTCCTA	CGGTCATGGGTCTTCACACA
*Plekhh1*	AGCCACGAGGACAAGAGAC	TCACAGGACCGATCTACTTCC
*Plp1*	TGAGCGCAACGGTAACAGG	TTCCCAAACAATGACACACCC
*Plp1 Ex2*–*4*	GGCCACTGGATTGTGTTTCT	GACTGACAGGTGGTCCAGGT
*Pstpip2*	GCACCATTGGCTACGACAG	ACACGGCTTCTTCCTGGAGA
*Sirt2*	GCCTGGGTTCCCAAAAGGAG	GAGCGGAAGTCAGGGATACC
*Smarcb1 Ex1*–*3*	ATTCTTTCCAGCTCGACCCC	CTTCTCGTCATTGCCATCCAG

### 2.7 RNA sequence analysis and data analysis

RNA sequencing (RNA-seq) was performed as previously described with some modifications (Bizen et al., 2022). Briefly, cerebral cortex were isolated from control mice and *Cnp-iCre*; *RelA* cKO mice at P14. Total RNA was extracted using the RNeasy Mini Kit (Qiagen). The integrity and quantity of the extracted RNA were assessed with an Agilent 2100 Bioanalyzer (Agilent Technologies). Subsequently, mRNA libraries were prepared following the Illumina TruSeq protocol, which involved polyA selection, fragmentation, and adapter ligation (TruSeq RNA Sample Preparation Kit v2, Illumina). The multiplexed libraries were sequenced as 150 nt paired-end reads on an Illumina NovaSeq 6,000 platform by Novogene company.^1^ The RNA-seq data was deposited as GSE294303 in the Gene Expression Omnibus (GEO) database. The sequencing reads were aligned to the reference genome (GRCm39/mm39) using STAR (Dobin et al., 2013), and gene expression levels were quantified with RSEM (Li and Dewey, 2011). Differential expression analysis was conducted using edgeR (Robinson et al., 2010), with a statistical threshold of FDR < 0.05 applied to determine significance. DAVID Bioinformatics Resources^2^ were used to perform Gene Ontology (GO) analysis (Huang et al., 2009). The threshold of GO analysis was set at *p* < 0.05. For comprehensive alternative splicing analysis, the sequence reads were re-aligned to the reference mouse genome (mm10) using OLego (Wu et al., 2013), and expression levels and alternative splicing events were quantified by Quantas^3^ The calculation of the exon inclusion rate and differential exon inclusion rate of each mRNA between two groups (Δ*I*) were statistically analyzed by Fisher exact test in the Quantas tool. The statistical criteria (*p* < 0.05, |Δ*I*| > 0.05) as significant changes of expression and alternative splicing was employed. To evaluate the enrichment of transcription factor binding motifs and the cell type specificity of gene expression, we utilized the Enrichr platform (Chen et al., 2013).^4^ Specifically, enrichment analyses were performed using TRANSFAC (Wingender et al., 2000) and JASPAR (Sandelin et al., 2004) databases for transcription factor binding motifs, and Cell Marker (Zhang et al., 2019) and Tabula Muris (Tabula Muris and Consortium, 2018) databases for cell type–specific gene expression profiles.

### 2.8 Cell culture for oligodendrocyte differentiation

Neural precursor cells (NPCs) were isolated and dissociated from the telencephalon of embryonic day 14.5 (E14.5) mice, and they were subsequently plated onto culture dishes pre-coated with poly-L-ornithine (Sigma-Aldrich) and fibronectin (Thermo Fisher Scientific). The cells were cultured in Dulbecco’s modified eagle medium/nutrient mixture (DMEM/F12) (pH 7.2, Invitrogen) supplemented with N2, which contained 25 μg/ml insulin (Sigma-Aldrich), 100 μg/mL apo-transferrin (Sigma-Aldrich), 20 nM progesterone (Sigma-Aldrich), 100 μM putrescine (Sigma-Aldrich), 30 nM sodium selenite (Sigma-Aldrich), and 1.27 mg/mL NaHO_3_ (Wako). Recombinant human fibroblast growth factor 2 (FGF2) (10 ng/mL, PeproTech) was also added to the medium to promote the proliferation of NPCs. The cells were maintained under these conditions for 4 days (Bizen et al., 2014, 2022). Following this initial culture period, the cells were dissociated and replated in DMEM/F12 medium supplemented with N2 (DMEM/F12/N2), FGF2, and recombinant murine PDGF-AA (10 ng/mL, PeproTech), and cultured for an additional 4 days to facilitate oligodendrocyte precursor cell (OPC) expansion. Subsequently, the cells were again dissociated and cultured for 5 days in DMEM/F12/N2 medium containing B27 supplement (Thermo Fisher Scientific), recombinant rat ciliary neurotrophic factor (CNTF) (10 ng/mL, PeproTech), triiodothyronine (T3, 30 ng/mL, Sigma), and thyroxine (T4, 40 ng/mL, Sigma) to induce differentiation into oligodendrocytes.

### 2.9 Western blotting analysis

Proteins were extracted from the cortex, corpus callosum and hippocampus at P14 in ice-cold RIPA buffer [50 mM Tris–HCl (pH 8.0), 150 mM NaCl, 1% NP–40, 0.5% sodium deoxycholate, 0.1% SDS, protease inhibitor cocktail (Complete Mini; Roche, Mannheim, Germany), and phosphatase inhibitor cocktail (PhosSTOP EASYpack; Roche)]. The lysates were sonicated by BioRuptor II (Cosmo Bio, Japan) and then centrifuged at 15,000 rpm for 15 min at 4°C. The supernatants were then collected. Protein concentrations were determined using BCA Protein assay kit (TaKaRa Bio, Japan). The samples were boiled in sodium dodecyl sulfate (SDS) sample buffer [2% SDS, 50 mM Tris–HCl (pH 6.8), 10% glycerol, 6% β-mercaptoethanol, 0.01% bromophenol blue] for 5 min at 95°C. The denatured lysates were separated by electrophoresis (24 mA for 75 min) in 5–20% SuperSep Ace agarose gels (Fujifilm Wako Pure Chemical Corp., Japan) and transferred to Hybond-P PVDF 0.45 (GE Healthcare, IL, United States; 250 mA for 100 min). Following blocking with 5% skim milk in TBST (25 mM Tris–HCl, 137 mM NaCl, 2.7 mM KCl, 0.05% Tween 20, finally adjusted to pH 7.5), the membrane was incubated with rabbit monoclonal anti-NF-κB p65 (D14E12) XP (1:1,000, #8242, Cell Signaling Technology) and mouse monoclonal anti-β-Actin (1:1,000, AC-15, Sigma) overnight at 4°C. After washing with TBST for 5 min three times, the membrane was reacted with the secondary antibodies conjugated to horseradish peroxidase (1:2,000, Cell Signaling Technology). Immunoreactivity was visualized by Western Lightning Plus-ECL enhanced chemiluminescence substrate (PerkinElmer, Massachusetts) or ImmunoStar LD (Fujifilm Wako Pure Chemical Corp.). Images were acquired using the C-DiGit blot scanner (LI-COR Biosciences, Lincoln, NE, United States). Signal intensities from immunoreactive bands were determined by densitometric measurement using ImageJ software.^5^

### 2.10 Statistical analyses

All experiments had at least three biological replications, unless otherwise stated. Data are reported as mean ± standard error of the mean (SEM). Histological analyses were conducted using an unpaired *t*-test to assess statistical significance. The *p*-value of less than 0.05 was considered statistically significant. All statistical analyses were performed using GraphPad Prism 9 software (Pinyomahakul et al., 2024).

## 3 Results

### 3.1 Generation of oligodendrocyte-targeted *RelA* cKO mice

**FIGURE 1:**
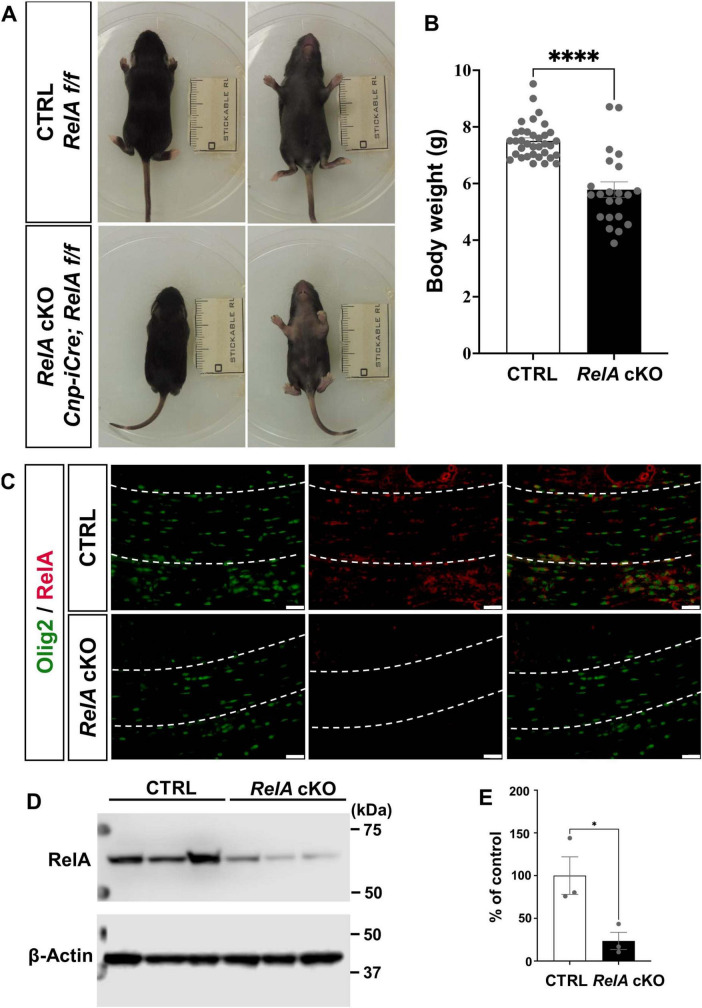
Generation of *RelA* conditional knockout (cKO) mice in oligodendrocytes. **(A)** Representative photographs showing growth retardation and hair loss in *Cnp-iCre*; *RelA* cKO mice at P14. **(B)** Bar graphs showing the average body weights of control (*n* = 35) and *Cnp-iCre*; *RelA* cKO (*n* = 22) mice. **(C)** Immunohistochemistry for RelA and Olig2, in the corpus callosum of control and *Cnp-iCre*; *RelA* cKO mice at P14 (*n* = 3 mice per group). The white dotted line indicates the region of the corpus callosum shown. **(D)** Western blotting for RelA and β-actin in Control and *RelA* cKO cerebral cortices at P14. β-actin was used as a loading control. **(E)** Quantification of relative RelA protein abundance was performed in control and *RelA* cKO mice, showing levels as a percentage of the control (*n* = 3 mice per group). Bar charts represent the mean ± SEM. Statistical analysis was performed by two-tailed, unpaired *t*-test. *p < 0.05; ****p < 0.0001. Scale bars, 40 μm **(C)**.

To investigate the role of RelA in oligodendrocyte lineage cells, we generated conditional knockout (cKO) mice by crossing female *Cnp-iCre*; *RelA*^*flox/*+^ mice with male *RelA^flox/flox^* mice. At postnatal day 14 (P14), *RelA* cKO mice exhibited significant growth retardation compared to control mice, as evidenced by a marked reduction in body weight (Figures 1A,B). In addition, the *RelA* cKO mice developed alopecia characterized by hair loss at this stage (Figure 1A). Previous studies have demonstrated that NF-κB signaling plays critical roles in Schwann cell differentiation and hair follicle development (Chen et al., 2011; Krieger et al., 2018). In addition, Cnp is known to be expressed not only in the central nervous system but also in peripheral Schwann cells and a subset of neural crest-derived cells (Yoshino et al., 1985; Iwashita et al., 2003), the observed growth delay and hair loss in *RelA* cKO mice may reflect the effects of *RelA* deficiency in the peripheral nervous system and skin.

To confirm the reduction of RelA protein expression in oligodendrocyte lineage cells of *RelA* cKO brain, we performed immunohistochemistry (IHC) for RelA and Oligodendrocyte transcription factor 2 (Olig2). The analysis revealed a marked reduction of RelA protein levels in Olig2-positive oligodendrocytes in the corpus callosum of *RelA* cKO mice (Figure 1C).

We performed Western blotting to further quantify the overall reduction of RelA protein in brain lysates at P14. The results demonstrate a substantial reduction of RelA protein in the cKO brain (Figures 1D,E).

### 3.2 Spatiotemporally restricted delay of oligodendrocyte differentiation in the *RelA* cKO mice

**FIGURE 2:**
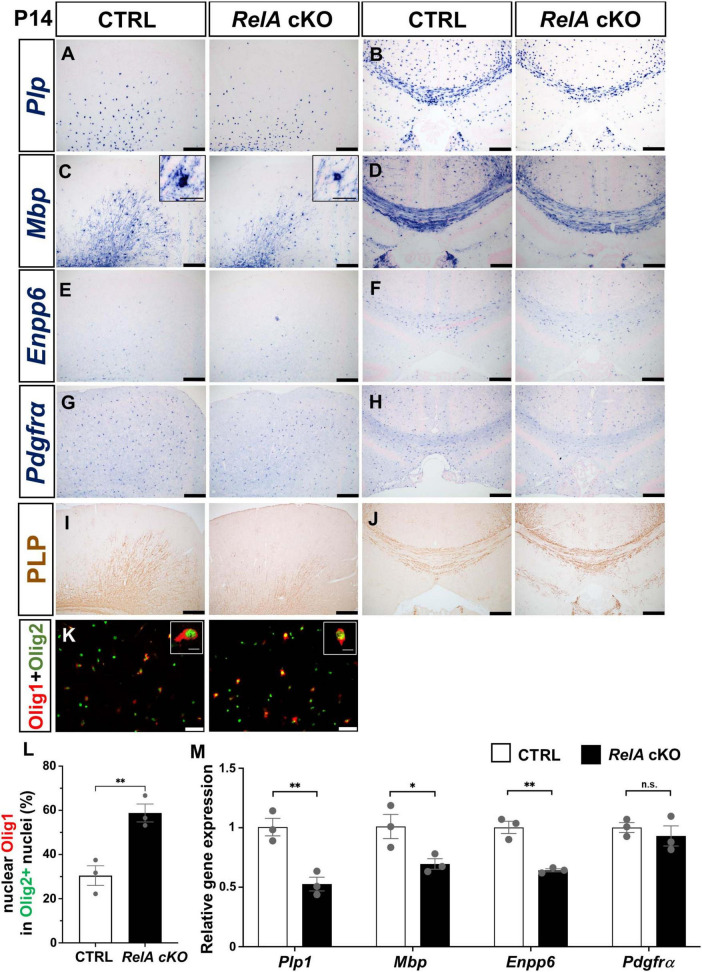
Transient delay of oligodendrocyte differentiation in *RelA*-deficient cerebral cortices. **(A–H)** *In situ* hybridization (ISH) showing reduced mRNA expression of mature oligodendrocyte markers *Plp* **(A,B)**, *Mbp* **(C,D)**, *Enpp6* **(E,F)**, and *Pdgfrα* **(G,H)** in the secondary motor cortex and corpus callosum of *RelA* cKO mice compared to controls at P14. **(I,J)** Immunohistochemistry (IHC) demonstrating decreased PLP protein levels in the motor cortex and corpus callosum of *RelA* cKO mice at P14. **(K)** Immunofluorescence (IF) showing Olig1 and Olig2 expression in the motor cortex of control and *RelA* cKO mice at P14. **(L)** Bar graph showing the percentage of Olig2-positive cells with nuclear localization of Olig1 in **(K)**. **(M)** RT-qPCR analysis of *Plp1*, *Mbp*, *Enpp6, and Pdgfrα* mRNA levels in mixed cortex, corpus callosum, and hippocampus from control and *RelA* cKO mice at P14. *n* = 3 mice per genotype for all experiments. Bar charts represent the mean ± SEM. Statistical analysis was performed by two-tailed, unpaired *t*-test. *p < 0.05; **p < 0.01; n.s., not significant. Scale bars, 200 μm **(A–J)**; 40 μm **(K)**. Inset scale bars, 10 μm **(K).**

To elucidate the role of RelA in oligodendrocyte differentiation, we examined the expression of oligodendrocyte-related genes in the cerebral cortex of *RelA*-deficient and control mice at P14 and P21 using *in situ* hybridization (ISH) and immunohistochemistry. At P14, *RelA*-deficient mice showed significantly reduced expression of myelin-related genes such as *Plp*, *Mbp*, and *Mog*, as well as newly differentiated oligodendrocyte markers *Enpp6* and *Gpr17*, particularly in the secondary motor cortex and in the corpus callosum (Figures 2A–F; Supplementary Figures 1A–D). In contrast, the expression level of the OPC marker *Pdgfrα* was not significantly different between *RelA* cKO mice and control mice (Figures 2G,H). Furthermore, PLP and CC1 protein levels were markedly decreased in the secondary motor cortex but not in the corpus callosum (Figures 2I,J; Supplementary Figures 1E,F). In addition, the number of cells exhibiting cytoplasmic localization of Olig1, a hallmark of oligodendrocyte maturation (Arnett et al., 2004), was significantly reduced (Figures 2K,L). Quantitative PCR (qPCR) also demonstrated a significant downregulation of oligodendrocyte marker genes in the cerebral cortex of *RelA* cKO mice, except *Pdgfrα* (Figure 2M). By P21, however, the differences in the expression of these genes and proteins were no longer apparent, and the subcellular localization of Olig1 was comparable between genotypes (Supplementary Figures 2, 3). Furthermore, ISH analysis of the spinal cords at both P14 and P21 revealed no notable differences in the expression of oligodendrocyte-related genes between *RelA*-deficient and control mice (Supplementary Figure 4). These results indicate that the impairment of oligodendrocyte differentiation caused by *RelA* deficiency is restricted to a specific developmental time window and regions within the cerebral cortex.

### 3.3 Cell-autonomous suppression of terminal differentiation in *RelA*-deficient oligodendrocyte progenitor cultures

**FIGURE 3:**
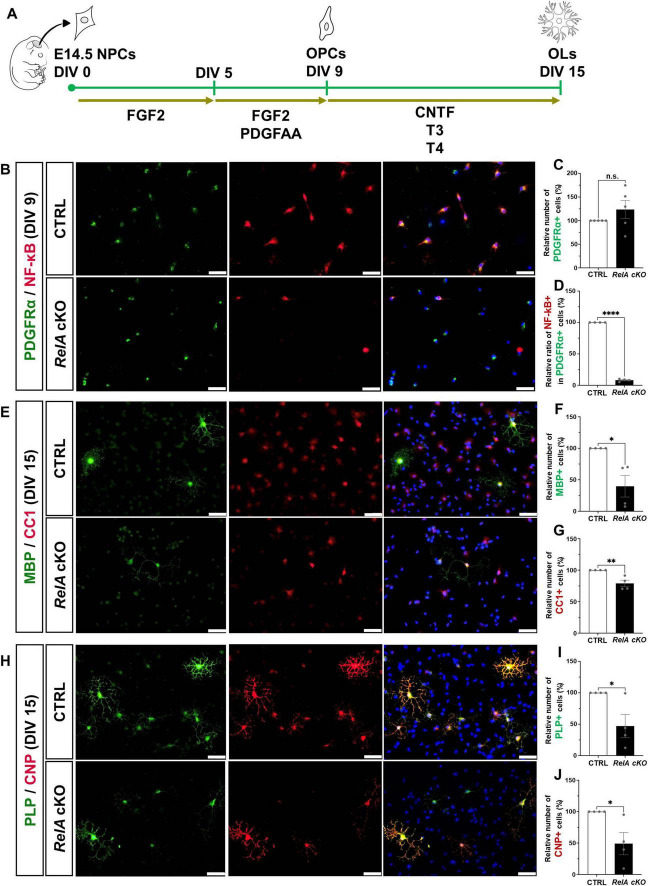
Cell-autonomous suppression of oligodendrocyte differentiation in *RelA*-deficient mice *in vitro*. **(A)** Schematic representation showing the experimental timeline of the primary culture for oligodendrocyte differentiation. **(B)** Immunofluorescence analysis of PDGFRα and RelA expression in OPCs derived from control and *RelA* cKO mice at 9 days *in vitro* (DIV 9). **(C,D)** Bar graphs represent the relative ratios of the number of PDGFRα-positive OPCs **(C)** or the proportion of RelA- and PDGFRα- double positive OPCs among PDGFRα-positive OPCs in *RelA* cKO mice **(D)** compared to control mice (*n* = 5 mice per group). **(E)** Immunofluorescence analysis of MBP and CC1 expression in mature oligodendrocytes derived from control and *RelA* cKO mice at DIV 15. **(F,G)** Bar graphs represent the relative ratios of the number of MBP- **(F)** or CC1-positive oligodendrocytes **(G)** in *RelA* cKO mice compared to control mice (*n* = 4 mice per group). **(H)** Immunofluorescence analysis of PLP and CNP expression in mature oligodendrocytes derived from control and *RelA* cKO mice at DIV 15. **(I,J)** Bar graphs represent the relative ratios of the number of PLP- **(I)** or CNP-positive oligodendrocytes **(J)** in *RelA* cKO mice compared to control mice (*n* = 4 mice per group). Bar charts represent the mean ± SEM. Statistical analysis was performed by two-tailed, unpaired *t*-test. *p < 0.05; **p < 0.01; ****p < 0.0001; n.s., not significant. Scale bars, 40 μm.

To determine whether the impaired oligodendrocyte differentiation observed in *RelA*-deficient mice is cell-autonomous, we utilized an oligodendrocyte differentiation-inducing culture system. Neural precursor cells (NPCs) were isolated from the telencephalon of E14.5 *RelA*-deficient and control embryos and expanded for 4 days in the presence of FGF2. These cells were then cultured for an additional 4 days with FGF2 and PDGF-AA, enriching for Olig2 and PDGFRα double-positive oligodendrocyte precursor cells (OPCs). Subsequent exposure to CNTF and thyroid hormones (T3 and T4) induced differentiation into oligodendrocytes (Figure 3A). Immunostaining confirmed a marked reduction of RelA protein in *RelA*-deficient OPCs prior to differentiation, although the number of OPCs was not significantly different from controls (Figures 3B–D). By day 5 of differentiation, the number of cells expressing multiple oligodendrocyte markers was significantly decreased in the *RelA*-deficient cultures (Figures 3E–J), indicating that loss of RelA intrinsically suppresses oligodendrocyte differentiation in a cell-autonomous manner.

### 3.4 Sustained activation of astrocytes and microglia in the cerebral cortex of *RelA* cKO mice

**FIGURE 4:**
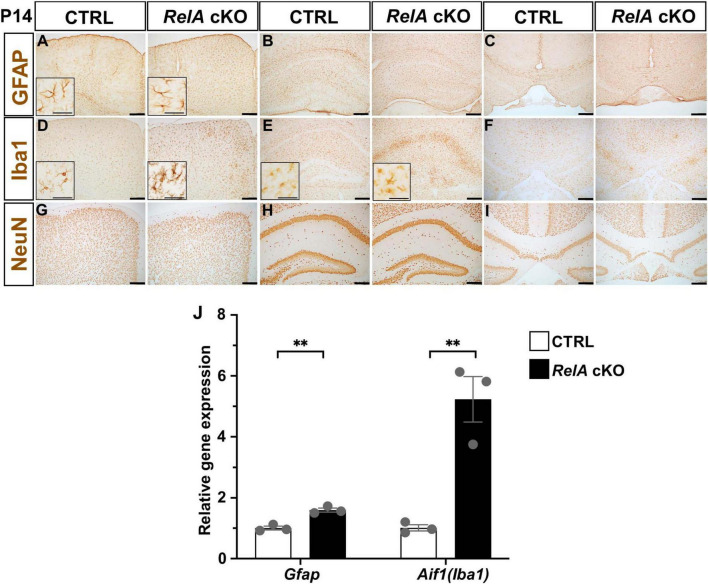
Activation of astrocytes and microglia in *RelA*-deficient cerebral cortices. **(A–C)** IHC for GFAP in the second motor cortex **(A)**, hippocampus **(B)**, and corpus callosum **(C)** of control and *RelA* cKO mice at P14. **(D–F)** IHC for Iba1 in the second motor cortex **(D)**, hippocampus **(E)**, and corpus callosum **(F)** of control and *RelA* cKO mice at P14. **(G–I)** IHC for NeuN in the second motor cortex **(G)**, hippocampus **(H)**, and corpus callosum **(I)** of control and *RelA* cKO mice at P14. **(J)** RT-qPCR analysis of *Gfap* and *Aif1* (*Iba1*) mRNA levels in cerebral cortices and hippocampi from control and *RelA* cKO mice at P14. *n* = 3 mice per genotype for all experiments. Bar charts represent the mean ± SEM. Statistical analysis was performed by two-tailed, unpaired *t*-test. **p < 0.01. Scale bars, 200 μm. Inset scale bars, 40 μm **(A,D,E)**.

To investigate whether *RelA* deficiency also affects other central nervous system (CNS) cell types, we performed immunohistochemical analyses on the brains of P14 mice. *RelA*-deficient mice exhibited a significant increase in the number of GFAP-positive astrocytes and Iba1-positive microglia, both showing morphological changes indicative of reactive gliosis in the cerebral cortex (Figures 4A,D; Supplementary Figure 5). In addition, an increase in microglial number and morphological alteration, but not those of astrocyte, was observed in the hippocampus (Figures 4B,E; Supplementary Figure 5), whereas no marked changes were detected in either astrocytes or microglia in the corpus callosum (Figures 4C,F; Supplementary Figure 5). In contrast, the number of NeuN-positive neurons remained largely unchanged (Figures 4G–I). qPCR analysis further confirmed a significant upregulation of *Gfap* and *Aif1* (*Iba1*) mRNA levels in the cerebral cortex of *RelA* cKO mice (Figure 4J). Notably, glial activation persisted at P21, even after the delay in oligodendrocyte differentiation had resolved (Supplementary Figure 6). These findings suggest that *RelA* deficiency in oligodendrocytes may contribute to continuous activation of surrounding glial cells in a non-cell-autonomous manner.

### 3.5 Transcriptomic analysis of the *RelA* cKO cerebral cortex

**FIGURE 5:**
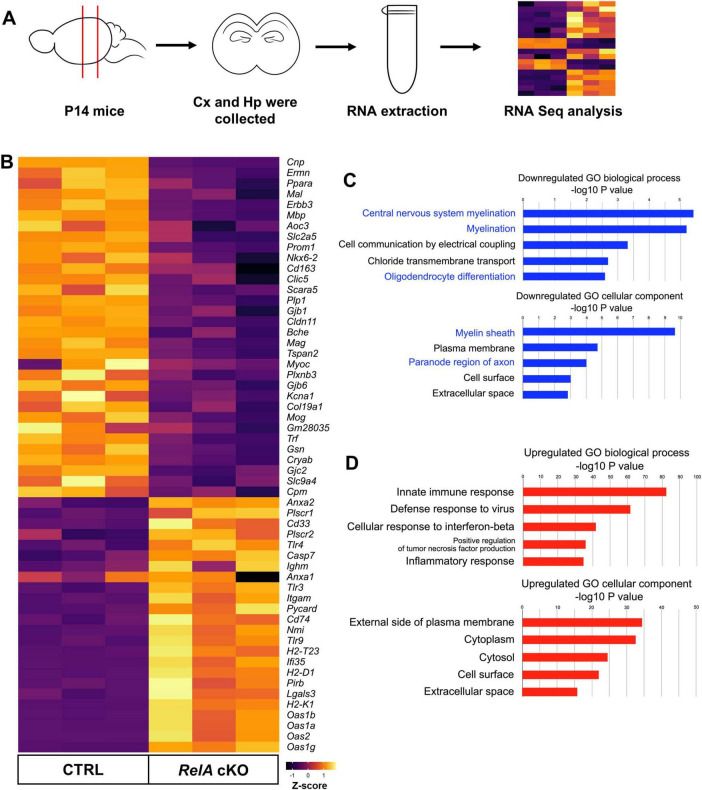
Transcriptomic analysis reveals downregulation of oligodendrocyte-related genes in *RelA*-deficient mice. **(A)** Schematic overview of the RNA extraction and RNA sequencing (RNA-seq) workflow. Cx, Cerebral cortex; Hp; Hippocampus. **(B)** Heatmap from RNA-seq data (*Z*–scores) depicts oligodendrocyte- and immune inflammatory response-related genes with significant differences in expression levels between control and *RelA* cKO mice. **(C)** Gene Ontology (GO) enrichment analysis of downregulated genes. Bar graphs illustrate the top five significantly enriched GO biological processes, demonstrating decreased representation of central nervous system myelination, myelination, and oligodendrocyte differentiation. Additionally, the top five significantly enriched GO cellular components show decreased representation of myelin sheath and paranode region of axon. **(D)** GO enrichment analysis of upregulated genes. Bar graphs illustrate the top five significantly enriched GO biological processes, demonstrating increased representation of immune response and inflammatory response. The top five significantly enriched GO cellular components show increased representation of external side of plasma membrane, cytoplasm, and cell surface.

To comprehensively characterize the transcriptional changes underlying the phenotypes observed in *RelA*-deficient mice, we performed RNA sequencing (RNA-seq) using RNA isolated from the cerebral cortex of *RelA*-deficient and control mice (Figure 5A). Differentially expressed genes (DEGs) were identified using stringent thresholds (|log_2_FC| > 0.58, FDR < 0.05), revealing 179 downregulated and 806 upregulated genes in the *RelA*-deficient cortex (Supplementary Table 1). Gene Ontology (GO) analysis of downregulated genes revealed significant enrichment for terms related to myelination and oligodendrocyte differentiation, whereas upregulated genes were associated with immune responses and inflammation (Figures 5B–D). These transcriptomic alterations support the notion that *RelA* deficiency impairs oligodendrocyte development while promoting glial activation, providing a molecular basis for the observed histological phenotypes.

### 3.6 Identification of putative RelA target genes involved in oligodendrocyte differentiation

**FIGURE 6:**
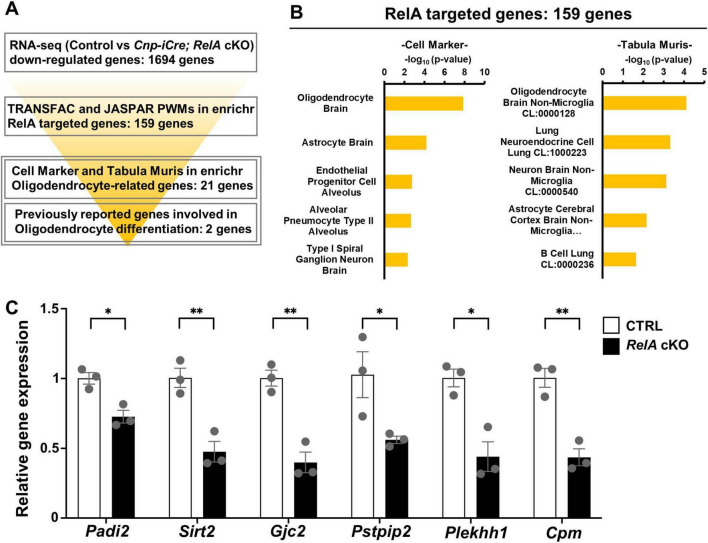
Identification of putative oligodendrocyte-related NF-κB RelA target genes. **(A)** Schematic illustration of the screening flow of putative oligodendrocyte-related NF-κB RelA-target genes. **(B)** Cell type–specific enrichment analysis of putative NF-κB RelA-targeted genes using Cell Marker and Tabula Muris. Enrichment analysis was performed on a set of potential NF-κB RelA target genes that were significantly downregulated in *RelA*-deficient mice (FDR < 0.05), using the Cell Marker (ver. 2024) and Tabula Muris datasets to evaluate their cell type–specific expression profiles. **(C)** RT-qPCR analysis of narrowed-down putative RelA-targeted genes related to oligodendrocyte. *n* = 3 mice per genotype. Bar charts represent the mean ± SEM. Statistical analysis was performed by two-tailed, unpaired *t*-test. *p < 0.05; **p < 0.01.

To elucidate the molecular mechanisms by which RelA regulates oligodendrocyte differentiation at the transcriptional level, we sought to identify potential direct targets of RelA among oligodendrocyte-related genes that were differentially expressed in the transcriptomic analysis of *RelA*-deficient mice (Figure 6A). We first performed motif enrichment analysis using the TRANSFAC and JASPAR databases available in the Enrichr platform on the set of genes significantly downregulated (FDR < 0.05) in *RelA*-deficient mice. This analysis yielded 159 genes predicted to contain RelA binding motifs. Next, to assess the cell-type specificity of these candidate genes, we conducted enrichment analyses using the Cell Marker and Tabula Muris datasets. These analyses revealed that terms associated with oligodendrocytes ranked highest, suggesting a strong enrichment of oligodendrocyte-related genes within the candidate set (Figure 6B). Among them, 21 genes were identified as being highly or specifically expressed in oligodendrocytes. In addition, two genes—peptidyl arginine deiminase2 (*Padi2*) (Falcão et al., 2019) and quaking (*Qk*) (Sidman et al., 1964; Darbelli et al., 2016; Zhou et al., 2020, 2021)—which have been previously reported to be involved in oligodendrocyte differentiation and maturation, were included, resulting in a final list of 23 putative RelA target genes (Supplementary Table 2). RT-qPCR analysis confirmed that several of these candidate genes, including *Padi2*, were significantly downregulated in the cerebral cortex of *RelA*-deficient mice (Figure 6C). These findings suggest that RelA may contribute to oligodendrocyte differentiation and maturation by directly regulating the transcription of a subset of lineage-specific genes essential for oligodendrocyte development.

### 3.7 Aberrant alternative splicing of oligodendrocyte-related genes in *RelA*-deficient mice

**FIGURE 7:**
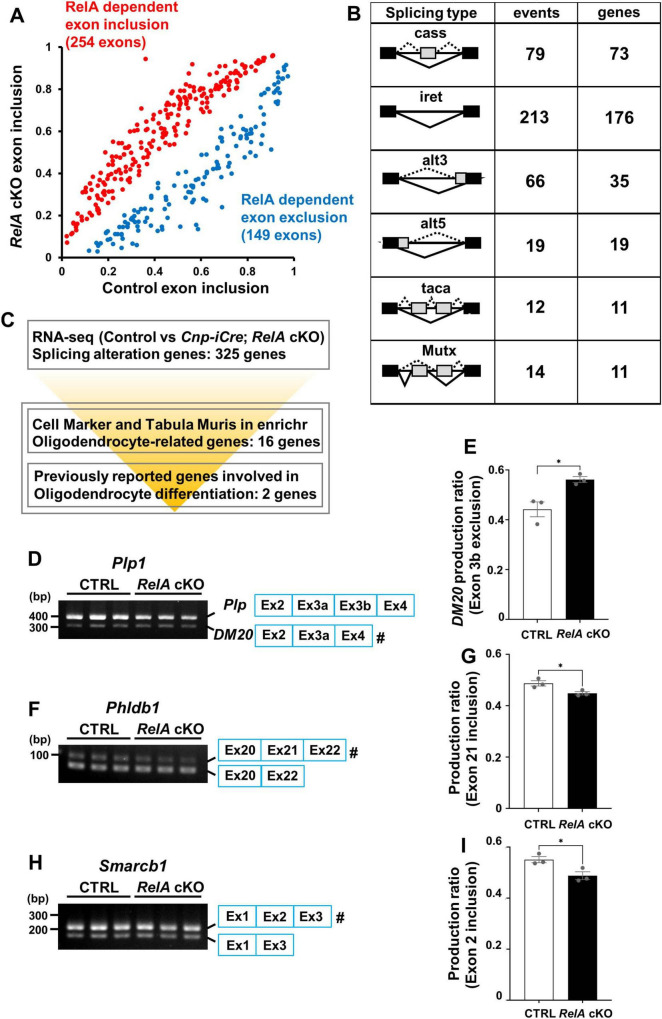
Alternative splicing alterations of oligodendrocyte-related genes in *RelA*-deficient mice. **(A)** Significant alternative splicing events identified from RNA–seq analysis of control and *RelA* cKO mice were visualized using a scatter plot based on percent spliced-in (PSI) values. Each data point represents the mean PSI value of an individual alternative splicing event, calculated from three independent biological replicates. **(B)** Diagram illustrating the patterns of alternative splicing dysregulation in cerebral cortices of *RelA* cKO mice. cass, cassette exon; taca, tandem cassette exon; Mutx, mutually exclusive exon; alt5, alternative 5’ site; alt3, alternative 3’ site; iret, intron retention. Black and gray boxes indicate the remaining exons and skipped exons, respectively, Solid and broken lines indicate the normal and abnormal splicing, respectively. The number of events or genes for splicing changes in *RelA* cKO mice is shown. **(C)** Overview of the screening workflow for alternative splicing alterations of oligodendrocyte-related genes in *RelA* cKO mice. **(D–I)** Semi-quantitative RT-PCR for the alternative splicing of *Plp1*, *Phldb1*, and *Smarcb1* mRNA in control and *RelA* cKO mice at P14. *n* = 3 mice per group. Bar charts show the ratio of specific exon exclusion (*Plp1* exon 3b, *DM20*) **(E)** or inclusion (*Phldb1* exon 21 and *Smarcb1* exon *2*) **(G,I)**. The hash key (#) indicates the band used for quantification: band density of the marked band was divided by the sum of densities for both bands (upper and lower bands). Bar charts represent the mean ± SEM. *p < 0.05. Signal intensities from electrophoretic bands were determined by densitometric measurement using ImageJ software.

In addition to its classical role as a transcription factor, members of the NF-κB family, including RelA, have been recently implicated in the regulation of RNA, such as alternative splicing (Ameur et al., 2020; Lee et al., 2020; Marie et al., 2024; Van Gelder et al., 2025). To explore this possibility, we analyzed exon junction usage in the RNA-seq data from the cerebral cortex of *RelA*-deficient and control mice. This analysis revealed 403 significant splicing alterations in *RelA*-deficient samples, comprising 149 exon skipping and 254 exon inclusion events (Figure 7A), categorized into six classes: cassette exons, mutually exclusive exons, tandem cassette exons, alternative 5′ site, alternative 3′ site, and intron retention (*p* < 0.05, |Δ*I*| > 0.05) (Figure 7B). Among these, we identified 18 oligodendrocyte-related genes exhibiting aberrant splicing (Figure 7C; Supplementary Table 3). Notably, *Plp1*, a major myelin protein component, showed altered splicing with increased amount of the shorter and embryo-predominant *DM20* isoform, as validated by semi-quantitative RT-PCR (Figures 7D,E). However, this splicing abnormality was resolved at P21 (Supplementary Figure 7). In addition, we also observed altered splicing with decreased amount of the longer isoforms of *Phldb1* and *Smarcb1* (Figures 7F–I). These results indicate that RelA contributes not only to transcriptional regulation but also to splicing control of gene essential for oligodendrocyte maturation (Ng et al., 2015) and gene associated with glioma risk (Viana-Pereira et al., 2020; Baskin et al., 2015).

## 4 Discussion

In this study, we found that RelA, a key subunit of the NF-κB transcription factor complex, plays a crucial role in regulating oligodendrocyte differentiation through the coordinated control of transcriptional and splicing mechanisms within specific temporal and spatial contexts. These findings encourage reevaluating the physiological significance of NF-κB signaling in oligodendrocyte differentiation, which has been previously underappreciated.

The involvement of NF-κB signaling in oligodendrocyte development and differentiation remains controversial, with conflicting findings reported in the literature. For example, clinical studies have shown that patients with copy number gains of the *IKBKG* gene (encoding NF-κB essential modulator, NEMO) exhibit reduced NF-κB activity alongside myelination defects (Philippe et al., 2013). Conversely, mouse model studies have demonstrated that activation of NF-κB signaling in the central nervous system (CNS) can protect oligodendrocytes from cell death and promote remyelination following demyelinating insults (Stone et al., 2017; Lei et al., 2020). On the other hand, a paucity of evidence indicates that inactivation of NF-κB signaling, including *RelA* deficiency, causes prominent oligodendrocyte abnormalities during normal development (Hilliard et al., 1999, 2002; van Loo et al., 2006; Raasch et al., 2011; Kretz et al., 2014). These weak loss-of-function phenotypes may be attributed to systemic or CNS-wide NF-κB knockout models. Because oligodendrocytes heavily rely on external signals from other neural cell types or cell-cell interactions during their differentiation and maturation. The global loss of NF-κB activity may result in a change in the surrounding microenvironment affecting oligodendrocytes. Therefore, phenotypes observed in nonspecific NF-κB-deficient models may not accurately reflect the cell-autonomous functions of NF-κB in oligodendrocytes. For instance, neuronal-derived factors such as neurotrophic signals, neurotransmitters, and electrical activity are known to promote OPC differentiation and myelination (Mount and Monje, 2017). Astrocyte-derived molecules, including trophic factors, GAP junction channels, thrombin inhibitors, and lipid supply, also contribute to oligodendrocyte differentiation, myelination, and remyelination (Camargo et al., 2017; Traiffort et al., 2020). Additionally, cytokines secreted by microglia [e.g., tumor necrosis factor alpha (TNFα), interleukin–1 beta (IL–1β), IL–6, interferon gamma (IFN-γ)] can either promote or inhibit OPC differentiation (Lombardi et al., 2019; Traiffort et al., 2020). In addition, reactive astrocytes secret negative regulators of oligodendrocyte differentiation, such as chemokines, adherent molecules, and proteoglycans (Traiffort et al., 2020). Thus, a complex physiological and pathological network of intercellular signals finely controls oligodendrocyte differentiation. Moreover, it is important to point out that compensation for transient delay in oligodendrocyte differentiation may occur at later stages. For example, IκBα-overexpressing mice were analyzed only at or after P21 (Stone et al., 2017). More refined analyses with spatiotemporal specificity are required to investigate the role of NF-κB signaling in the oligodendrocyte differentiation.

In this study, by employing *RelA* cKO mice in oligodendrocytes, we could directly evaluate the role of RelA in oligodendrocyte differentiation. Our results demonstrated that oligodendrocyte-specific *RelA* deficiency led to delayed differentiation, but this effect was limited in both its temporal window and affected brain regions. The oligodendrocyte phenotype was more pronounced *in vitro*, whereas *in vivo* changes were relatively modest. One possible explanation for this discrepancy is that other cell types, such as astrocytes and microglia, may partially compensate for the oligodendrocyte defect *in vivo* as described above. Because the timing and rate of oligodendrocyte differentiation vary across brain and spinal cord regions (Ozarkar et al., 2025), it is also possible that region-specific oligodendrocyte phenotypes were observed at the timing of analysis at P14, and then recovery occurred later stage at P21. As a third possible explanation, differences in the distribution of oligodendrocyte subtypes may underlie the different requirements for RelA in oligodendrocyte differentiation. Recent single-cell transcriptomic studies have classified mature oligodendrocytes into six distinct subgroups (MOL1–MOL6), each characterized by unique gene expression signatures (Marques et al., 2016).^6^ In these datasets, *RelA* expression is relatively high in MOL1, MOL2, and MOL6 and lower in MOL3–MOL5. Notably, in the corpus callosum of juvenile mice—where differentiation defects were evident in *RelA* cKO brain—MOL1 was predominant, whereas in the spinal dorsal horn and the somatosensory cortex—where no significant impairment was observed—MOL2–MOL5 were more prevalent. Although the distribution of oligodendrocyte subgroups in the secondary motor cortex remains unclear, it is plausible that regional differences in the abundance of RelA-high *versus* RelA-low subtypes influence the severity of the phenotypic outcome upon *RelA* deletion.

Several mechanisms are known to regulate the timing of oligodendrocyte differentiation. Nkx2.2 modulates the transition timing from OPCs to mature oligodendrocytes by directly regulating *Pdgfra* expression (Zhu et al., 2014). However, *Pdgfra* expression was not significantly affected by *RelA* deletion. Our RNA-seq data revealed a significant reduction in *Padi2* expression, suggesting that *Padi2* is one of the direct RelA-target genes. *Padi2* encodes peptidyl arginine deiminase 2 (PAD2), which post-translationally modifies various proteins, including MBP (Wood et al., 2008). *Padi2* cKO mice show a transient delay in oligodendrocyte differentiation during early postnatal stages, followed by normalization (Falcão et al., 2019), similar to *RelA* cKO mice. These findings support the possibility that a RelA–Padi2 axis plays a crucial role in controlling the timing of oligodendrocyte differentiation. Various upstream stimuli activate NF-κB, including pro-inflammatory cytokines such as IL-1β, TNFα, and IL-6, as well as developmental toolkit pathways like Notch and Wnt (Guo et al., 2024). Some factors promote oligodendrocyte differentiation, while others act as inhibitors (Wang et al., 1998; John et al., 2002; Shimizu et al., 2005; Fancy et al., 2009; Feigenson et al., 2009; Dai et al., 2014; Tran et al., 2023). Therefore, NF-κB may thus function as an integrative effector that reconciles these opposing signals to fine-tune oligodendrocyte development. This concept further supports the hypothesis that NF-κB/RelA plays a key role in regulating the timing of oligodendrocyte differentiation. We performed an *in silico* analysis based on known RelA-binding motifs to identify candidate oligodendrocyte-related target genes. However, whether these genes are direct RelA targets during oligodendrocyte differentiation remains to be determined. Although previous ChIP-seq analyses using cultured mouse astrocytes reported several candidate genes with high RelA-binding scores, including *Sirt2* and *Phldb1* (Nakano-Kobayashi et al., 2023),^7^ chromatin structure and accessibility for transcription factor in each locus vary significantly depending on the cell type and differentiation state, which limits the direct applicability of the data to oligodendrocyte lineage.

Our splicing analysis revealed that *RelA* deficiency resulted in aberrant splicing of several genes, such as *Plp1*, *Phldb1*, and *Smarcb1*. Interestingly, recent studies have shown that RelA also participates in alternative splicing regulation in addition to its canonical transcriptional activity (Ameur et al., 2020; Lee et al., 2020; Marie et al., 2024; Van Gelder et al., 2025). Specifically, RelA interacts with the splicing regulatory factor DEAD box protein 17 (DDX17) in an NF-κB-dependent manner, thereby modulating exon selection through the RNA helicase activity of DDX17 (Ameur et al., 2020). Notably, *Plp1* splicing defects have also been observed in oligodendrocytes lacking Ddx20 (Simankova et al., 2021), raising the possibility that RelA and Ddx20 may functionally cooperate in the regulation of *Plp1* splicing.

Continued activation of microglia and astrocytes was observed until postnatal day 21 (P21). However, oligodendrocyte differentiation appeared to recover at this stage, suggesting the persistence of subtle abnormalities in the *RelA* cKO brain. These observations provide important insights into the underlying neuropathology. Although oligodendrocyte differentiation was primarily evaluated based on the expression of lineage-specific markers, normalization of marker expression does not necessarily imply complete restoration of the ultrastructural integrity of myelin. Indeed, in *Cnp*-deficient mice, ultrastructural defects in myelin and axonal degeneration have been reported despite the absence of overt abnormalities in major myelin protein expression and were accompanied by microglial and astrocytic activation (Lappe-Siefke et al., 2003). Therefore, the sustained activation of microglia and astrocytes in *RelA*-deficient mice may reflect subtle and chronic disturbances in myelin architecture and axonal homeostasis, potentially due to abnormalities in *Plp1/DM20* splicing (Stecca et al., 2000; Wang et al., 2008), highlighting the necessity of further ultrastructural and morphological analyses.

In conclusion, this study identifies RelA as a novel regulatory factor involved in the temporal control of oligodendrocyte differentiation. The precise timing of oligodendrocyte differentiation is essential for the proper formation and functional integration of neural circuits. Indeed, a transient delay in oligodendrocyte differentiation has also been reported in mice lacking discoidin domain receptor 1 (Ddr1), which exhibit neurological deficits such as reduced locomotor activity, muscle weakness, and impaired motor coordination (Mei et al., 2023). These findings suggest that the delayed oligodendrocyte differentiation observed in the cerebral cortex of *RelA*-deficient mice may similarly contribute to neural dysfunction.

## Data Availability

The datasets presented in this study can be found in online repositories. The names of the repository/repositories and accession number(s) can be found in this article, in the “Materials and methods” section.

## Footnotes

1
https://en.novogene.com/

2
https://david.ncifcrf.gov/summary.jsp

3
https://zhanglab.c2b2.columbia.edu/index.php/Quantas

4
https://maayanlab.cloud/Enrichr/

5
https://imagejnihgov/ij/

6
https://linnarssonlab.org/oligodendrocytes/

7
https://chip-atlas.org

## Supplementary material

Supplementary Figure 1 Reduced expression of oligodendrocyte-related markers in the motor cortex and corpus callosum of *RelA* cKO mice at P14. **(A–D)** ISH analysis of *Mog* and *Gpr17* mRNA expression in the secondary motor cortex and corpus callosum of control and *RelA* cKO mice at P14. **(E,F)** IHC analysis of CC1 expression in the secondary motor cortex and corpus callosum of control and *RelA* cKO mice at P14. **(G)** RT-qPCR analysis of *Mog* and *Gpr17* mRNA levels in the cerebral cortex including hippocampus of control and *RelA* cKO mice at P14. *n* = 3 mice per genotype for all experiments. Bar charts represent the mean ± SEM. Statistical analysis was performed by two-tailed, unpaired *t*-test. **p* < 0.05; ^**^*p* < 0.01. Scale bars, 200 μm. Inset scale bars, 40 μm **(A)**.

Supplementary Figure 2 Recovery from delayed oligodendrocyte differentiation in the secondary motor cortex and corpus callosum of *RelA*-deficient mice at P21. **(A–L)** ISH analysis of *Plp, Mbp, Mog, Gpr17, Enpp6*, and *Pdgfrα* mRNA expression in the secondary motor cortex **(A,C,E,G,I,K)** and corpus callosum **(B,D,F,H,J,L)** of control and *RelA* cKO mice at P21. **(M–P)** IHC analysis of PLP and CC1 expression in the secondary motor cortex **(M,O)** and corpus callosum **(N,P)** of control and *RelA* cKO mice at P21. **(Q)** Immunofluorescence analysis of Olig1 and Olig2 expression in the secondary motor cortex of control and *RelA* cKO mice at P21. *n* = 3 mice per genotype for all experiments. Scale bars, 200 μm **(A–P)**; 40 μm, Inset scale bars, 10 μm **(Q)**.

Supplementary Figure 3 RT-qPCR analysis of oligodendrocyte-related markers in the cerebral cortex including hippocampus of *RelA* cKO mice at P21. RT–qPCR analysis of *Plp, Mbp, Mog, Gpr17, Enpp6, Olig2*, and *Olig1* mRNA levels in the cerebral cortex including hippocampus of control and *RelA* cKO mice at P21. *n* = 3 mice per genotype. Bar charts represent the mean ± SEM. Statistical analysis was performed by two-tailed, unpaired *t*-test. n.s., not significant.

Supplementary Figure 4 Unaltered oligodendrocyte differentiation in the spinal cords of *RelA* cKO mice. **(A–F)** *In situ* hybridization (ISH) showing reduced mRNA expression of mature oligodendrocyte markers *Mbp* **(A,D)**, *Plp* **(B,E)**, *Mog* **(C,F)**, in the spinal cords of *RelA* cKO mice compared to controls at P14 **(A–C)** and P21 **(D–F)**. *n* = 3 mice per genotype. Scale bars, 200 μm.

Supplementary Figure 5 Resional microglia activation in *RelA*-deficient mice at P14. Iba1 IHC in the coronal sections of control and *RelA* cKO brains at P14 (*n* = 3 mice per group). Scale bar, 500 μm.

Supplementary Figure 6 Activation of astrocytes and microglia in *RelA*-deficient mice at P21. **(A–C)** IHC analysis of GFAP in the secondary motor cortex **(A)**, hippocampus **(B)**, and corpus callosum **(C)** of control and *RelA* cKO mice at P21. **(D–F)** IHC analysis of Iba1 in the secondary motor cortex **(D)**, hippocampus **(E)**, and corpus callosum **(F)** of control and *RelA* cKO mice at P21. **(G–I)** IHC analysis of NeuN in the secondary motor cortex **(G)**, hippocampus **(H)**, and corpus callosum **(I)** of control and *RelA* cKO mice at P21. **(J)** RT-qPCR analysis of *Gfap* and *Aif1* (*Iba1*) mRNA levels in the cerebral cortex including hippocampus of *RelA* cKO mice compared to controls at P21. *n* = 3 mice per genotype for all experiments. Bar charts represent the mean ± SEM. Statistical analysis was performed by two-tailed, unpaired *t*-test. **p* < 0.05; n.s., not significant. Scale bars, 200 μm. Inset scale bars, 40 μm.

Supplementary Figure 7 Resolution of *Plp1* splicing abnormalities in *RelA* cKO mice at P21. Semi-quantitative RT-PCR for the alternative splicing of *Plp1* mRNA in control and *RelA* cKO mice at P21. *n* = 3 mice per group. Bar charts show the ratio of specific exon inclusion or exclusion in each mRNA. Bar charts represent the mean ± SEM. n.s., not significant. Signal intensities from electrophoretic bands were determined by densitometric measurement using ImageJ software.
