# Supplementary figures and images for: NF-κB RelA regulates temporal oligodendrocyte differentiation in the postnatal brains
Source: Front Cell Neurosci. 2025 Jul 22;19:1622874. doi: 10.3389/fncel.2025.1622874 (PMC12321842; doi:10.3389/fncel.2025.1622874)

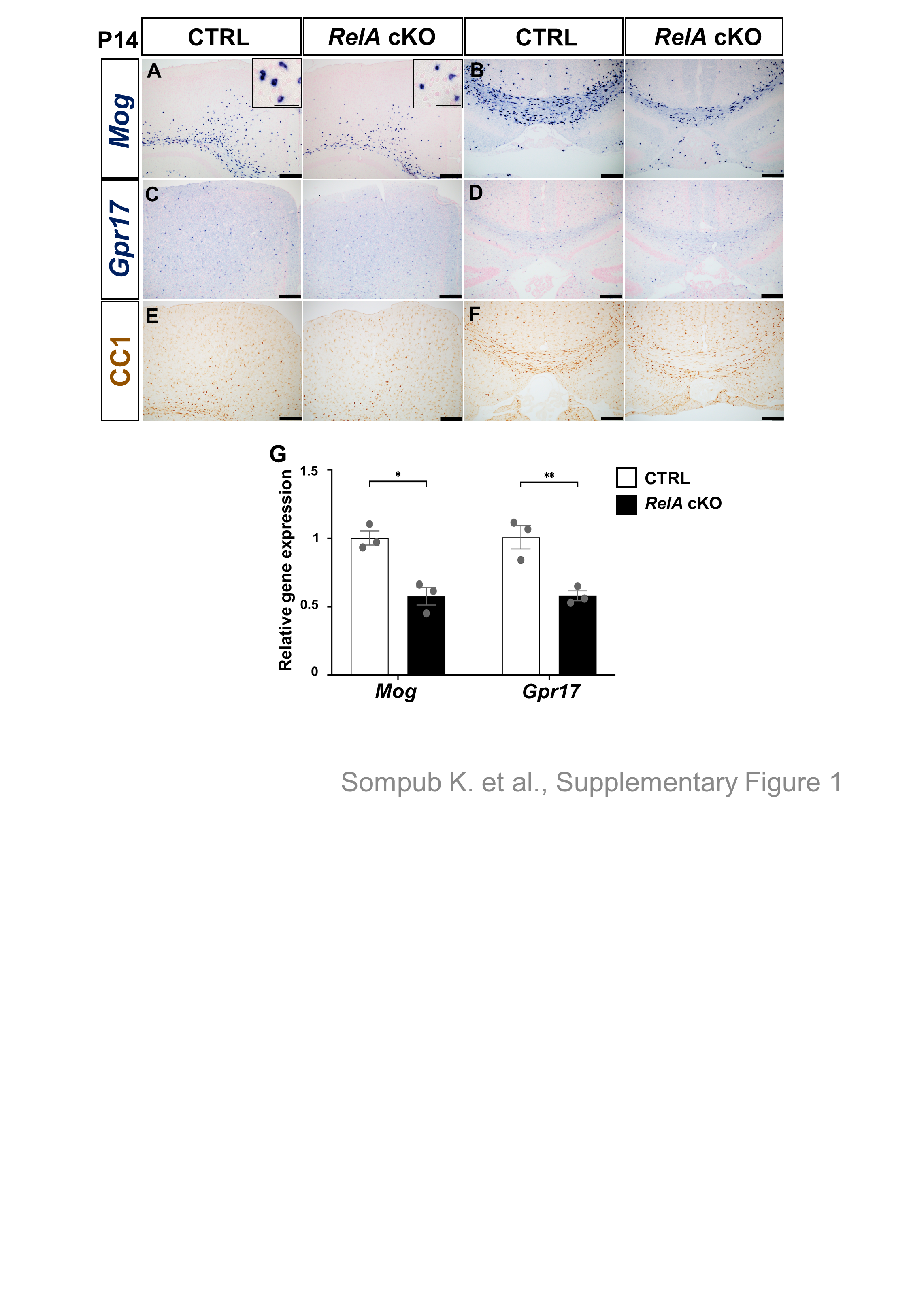

Supplement: Supplementary Figure 1 — Reduced expression of oligodendrocyte-related markers in the motor cortex and corpus callosum of RelA cKO mice at P14. (A–D) ISH analysis of Mog and Gpr17 mRNA expression in the secondary motor cortex and corpus callosum of control and RelA cKO mice at P14. (E,F) IHC analysis of CC1 expression in the secondary motor cortex and corpus callosum of control and RelA cKO mice at P14. (G) RT-qPCR analysis of Mog and Gpr17 mRNA levels in the cerebral cortex including hippocampus of control and RelA cKO mice at P14. n = 3 mice per genotype for all experiments. Bar charts represent the mean ± SEM. Statistical analysis was performed by two-tailed, unpaired t-test. *p < 0.05; **p < 0.01. Scale bars, 200 μm. Inset scale bars, 40 μm (A). [file Image_1.tif]

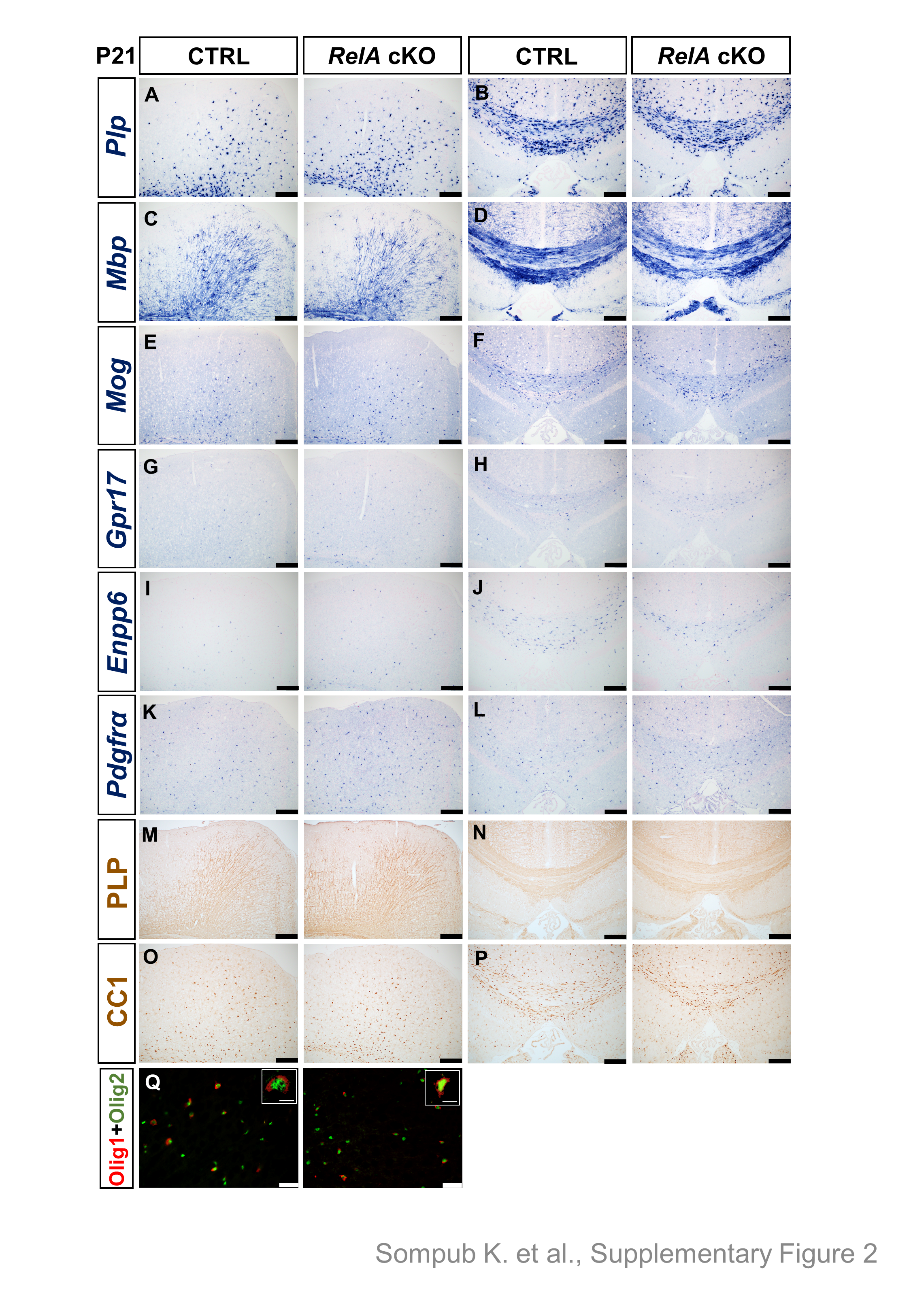

Supplement: Supplementary Figure 2 — Recovery from delayed oligodendrocyte differentiation in the secondary motor cortex and corpus callosum of RelA-deficient mice at P21. (A–L) ISH analysis of Plp, Mbp, Mog, Gpr17, Enpp6, and Pdgfrα mRNA expression in the secondary motor cortex (A,C,E,G,I,K) and corpus callosum (B,D,F,H,J,L) of control and RelA cKO mice at P21. (M–P) IHC analysis of PLP and CC1 expression in the secondary motor cortex (M,O) and corpus callosum (N,P) of control and RelA cKO mice at P21. (Q) Immunofluorescence analysis of Olig1 and Olig2 expression in the secondary motor cortex of control and RelA cKO mice at P21. n = 3 mice per genotype for all experiments. Scale bars, 200 μm (A–P); 40 μm, Inset scale bars, 10 μm (Q). [file Image_2.tif]

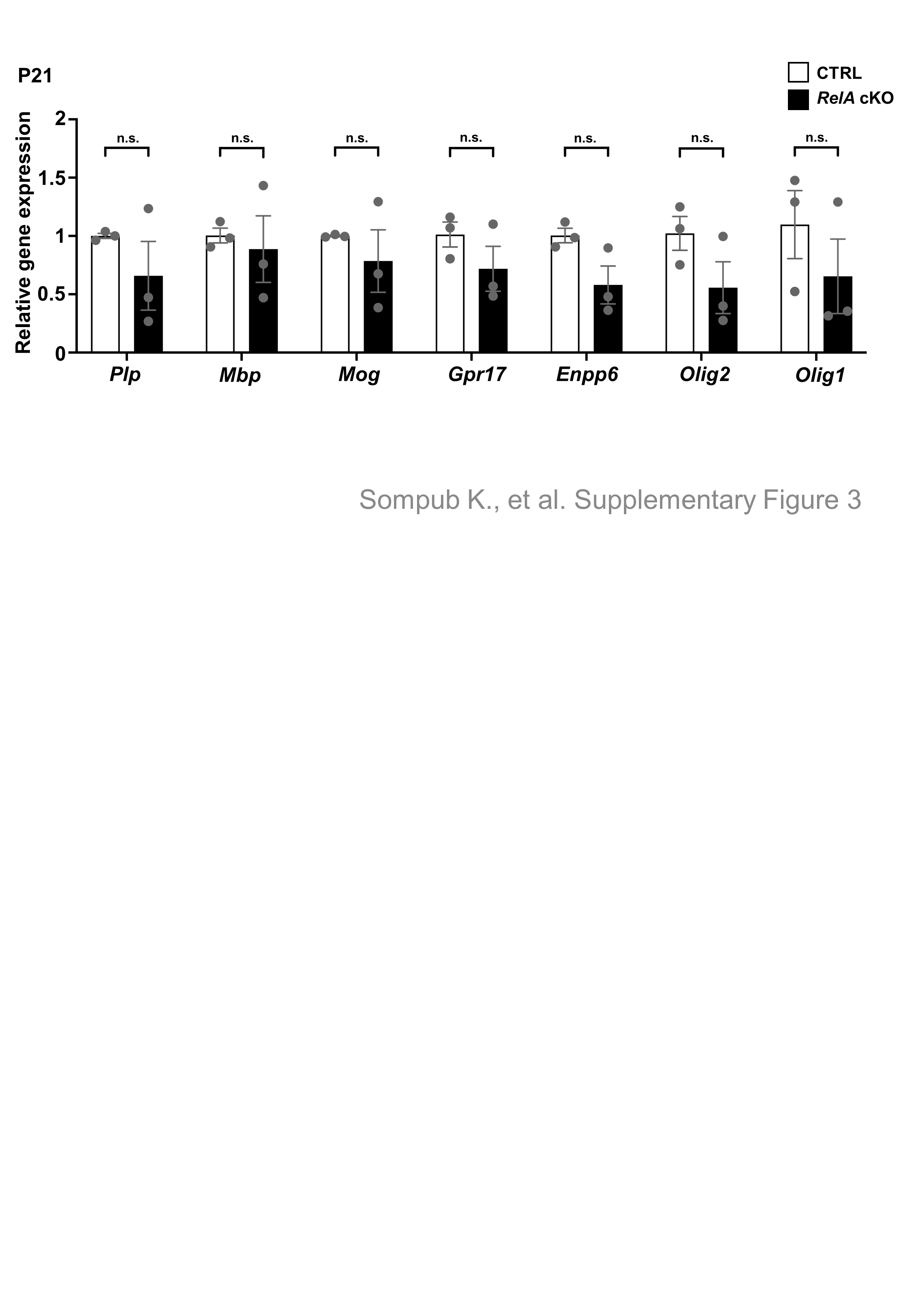

Supplement: Supplementary Figure 3 — RT-qPCR analysis of oligodendrocyte-related markers in the cerebral cortex including hippocampus of RelA cKO mice at P21. RT–qPCR analysis of Plp, Mbp, Mog, Gpr17, Enpp6, Olig2, and Olig1 mRNA levels in the cerebral cortex including hippocampus of control and RelA cKO mice at P21. n = 3 mice per genotype. Bar charts represent the mean ± SEM. Statistical analysis was performed by two-tailed, unpaired t-test. n.s., not significant. [file Image_3.tif]

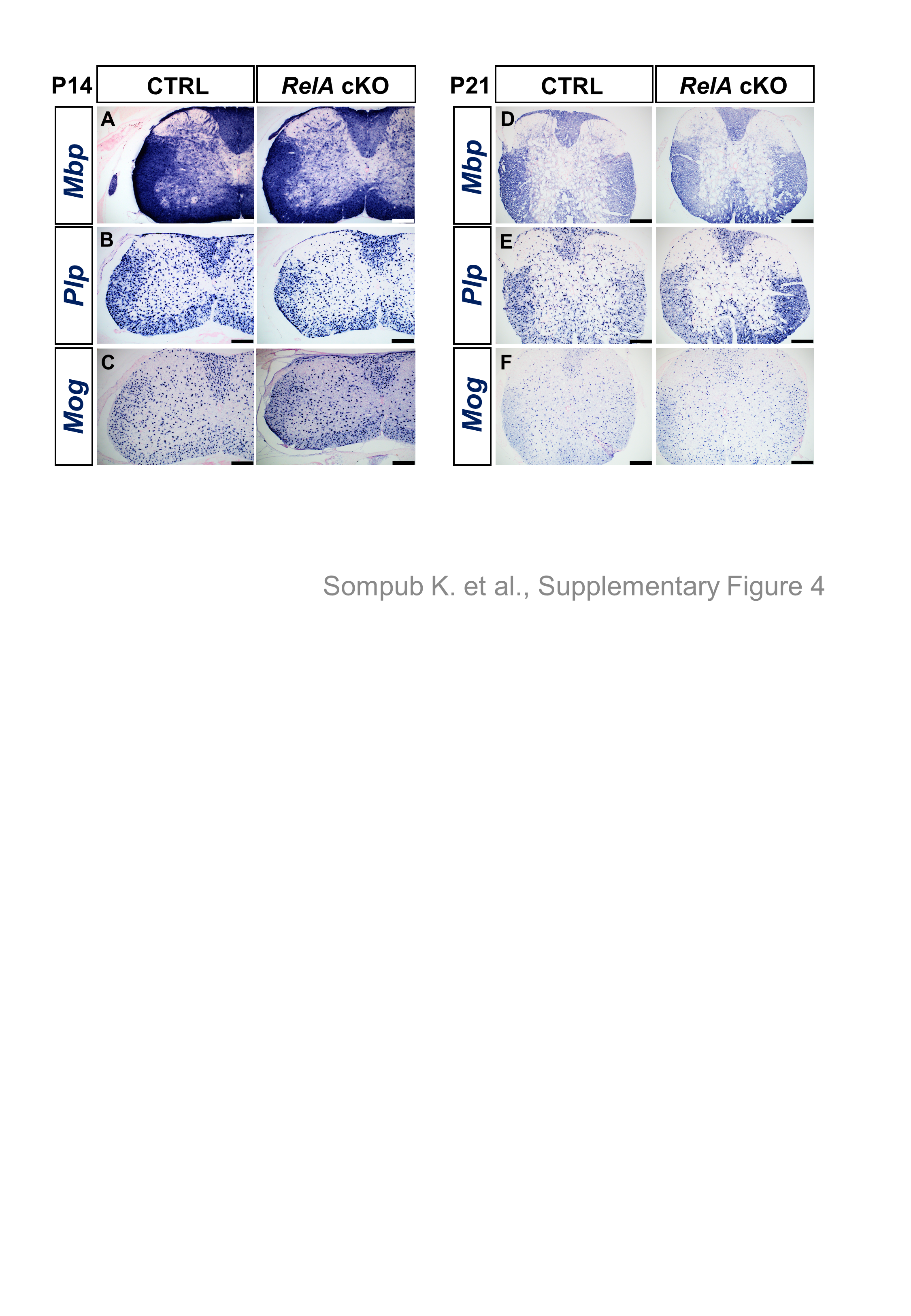

Supplement: Supplementary Figure 4 — Unaltered oligodendrocyte differentiation in the spinal cords of RelA cKO mice. (A–F) In situ hybridization (ISH) showing reduced mRNA expression of mature oligodendrocyte markers Mbp (A,D), Plp (B,E), Mog (C,F), in the spinal cords of RelA cKO mice compared to controls at P14 (A–C) and P21 (D–F). n = 3 mice per genotype. Scale bars, 200 μm. [file Image_4.tif]

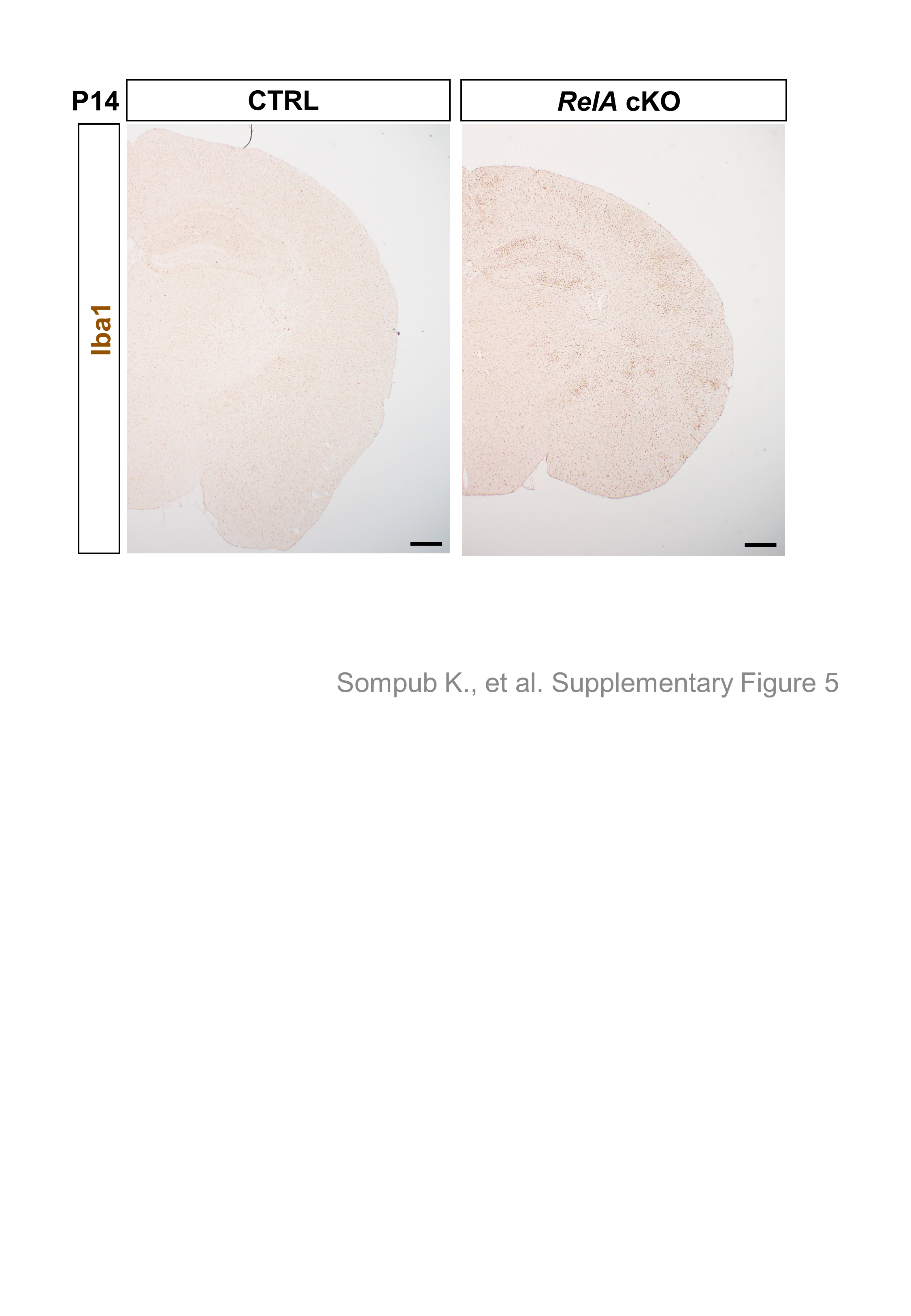

Supplement: Supplementary Figure 5 — Resional microglia activation in RelA-deficient mice at P14. Iba1 IHC in the coronal sections of control and RelA cKO brains at P14 (n = 3 mice per group). Scale bar, 500 μm. [file Image_5.tif]

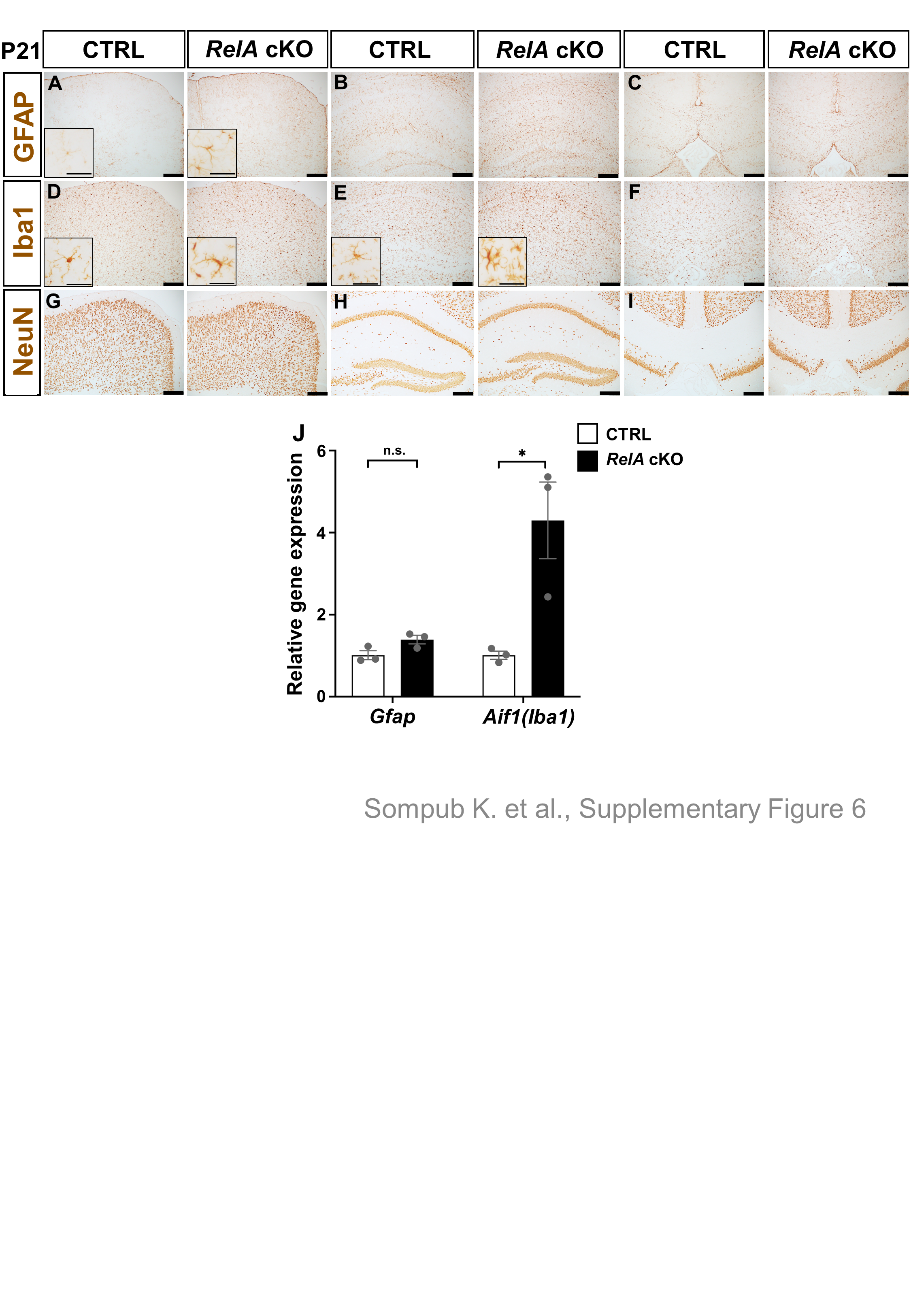

Supplement: Supplementary Figure 6 — Activation of astrocytes and microglia in RelA-deficient mice at P21. (A–C) IHC analysis of GFAP in the secondary motor cortex (A), hippocampus (B), and corpus callosum (C) of control and RelA cKO mice at P21. (D–F) IHC analysis of Iba1 in the secondary motor cortex (D), hippocampus (E), and corpus callosum (F) of control and RelA cKO mice at P21. (G–I) IHC analysis of NeuN in the secondary motor cortex (G), hippocampus (H), and corpus callosum (I) of control and RelA cKO mice at P21. (J) RT-qPCR analysis of Gfap and Aif1 (Iba1) mRNA levels in the cerebral cortex including hippocampus of RelA cKO mice compared to controls at P21. n = 3 mice per genotype for all experiments. Bar charts represent the mean ± SEM. Statistical analysis was performed by two-tailed, unpaired t-test. *p < 0.05; n.s., not significant. Scale bars, 200 μm. Inset scale bars, 40 μm. [file Image_6.tif]

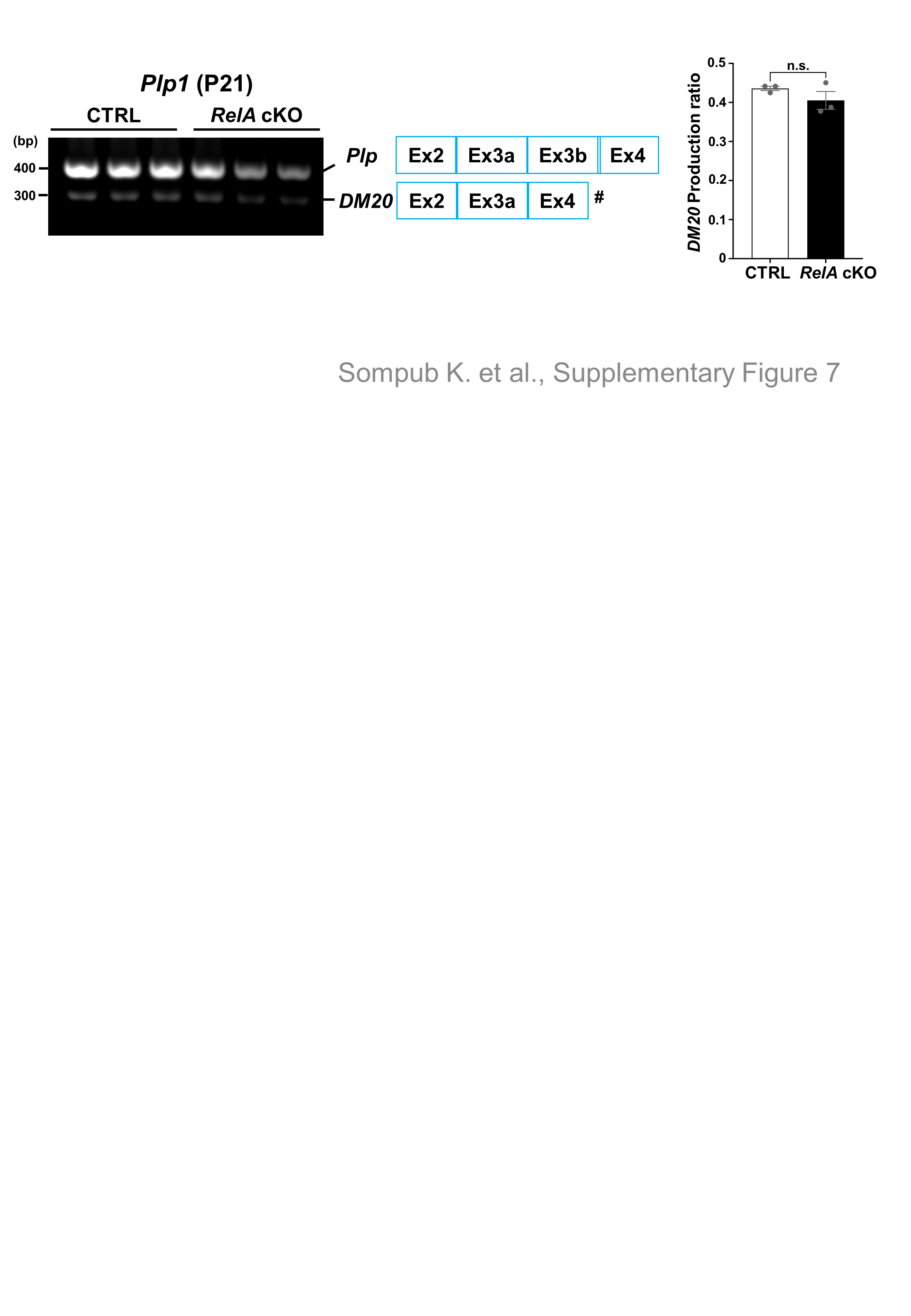

Supplement: Supplementary Figure 7 — Resolution of Plp1 splicing abnormalities in RelA cKO mice at P21. Semi-quantitative RT-PCR for the alternative splicing of Plp1 mRNA in control and RelA cKO mice at P21. n = 3 mice per group. Bar charts show the ratio of specific exon inclusion or exclusion in each mRNA. Bar charts represent the mean ± SEM. n.s., not significant. Signal intensities from electrophoretic bands were determined by densitometric measurement using ImageJ software. [file Image_7.tif]
